# Normative ranges of biventricular volumes and function in healthy term newborns

**DOI:** 10.1186/s12968-023-00932-1

**Published:** 2023-04-24

**Authors:** Simone Jhaveri, Ellie Battersby, Kenan W. D. Stern, Jennifer Cohen, Yang Yang, Anthony Price, Emer Hughes, Lucilla Poston, Dharmintra Pasupathy, Paul Taylor, Matias C. Vieira, Alan Groves

**Affiliations:** 1 Department of Pediatric Cardiology, Icahn School of Medicine at Mount Sinai, New York, NY USA; 2 Zucker School of Medicine at Hofstra/Northwell, Cohen Children’s Medical Center of New York, New Hyde Park, NY USA; 3 Center for the Developing Brain, Kings College London, London, UK; 4 Biomedical Engineering and Imaging Institute, Icahn School of Medicine at Mount Sinai, New York, NY USA; 5 Department of Women and Children’s Health, School of Life Course and Population Sciences, Kings College London, London, UK; 6 Reproduction and Perinatal Centre, Faculty of Medicine and Health, University of Sydney, Syndey, NSW Australia; 7 Department of Pediatrics, Dell Medical School at the University of Austin, Austin, TX USA

**Keywords:** Neonates, Cardiovascular magnetic resonance imaging, Normative data, Left ventricular volume, Left ventricular mass, Right ventricular volume, Right ventricular mass

## Abstract

**Background:**

Cardiovascular magnetic resonance (CMR) is increasingly used in newborns with congenital heart disease. However, reporting on ventricular volumes and mass is hindered by an absence of normative data in this population.

**Design/methods:**

Healthy term (37–41 weeks gestation) newborns underwent non-sedated, free-breathing CMR within the first week of life using the ‘feed and wrap’ technique. End-diastolic volume (EDV), end-systolic volume (ESV) stroke volume (SV) and ejection fraction (EF) were calculated for both left ventricle (LV) and right ventricle (RV). Papillary muscles were separately contoured and included in the myocardial volume. Myocardial mass was calculated by multiplying myocardial volume by 1.05 g/ml. All data were indexed to weight and body surface area (BSA). Inter-observer variability (IOV) was performed on data from 10 randomly chosen infants.

**Results:**

Twenty healthy newborns (65% male) with a mean (SD) birth weight of 3.54 (0.46) kg and BSA of 0.23 (0.02) m2 were included. Normative LV parameters were indexed EDV 39.0 (4.1) ml/m^2^, ESV 14.5 (2.5) ml/m^2^ and ejection fraction (EF) 63.2 (3.4)%. Normative RV indexed EDV, ESV and EF were 47.4 (4.5) ml/m^2^, 22.6 (2.9) ml/m^2^ and 52.5 (3.3)% respectively. Mean LV and RV indexed mass were 26.4 (2.8) g/m^2^ and 12.5 (2.0) g/m^2^, respectively. There was no difference in ventricular volumes by gender. IOV was excellent with an intra-class coefficient > 0.95 except for RV mass (0.94).

**Conclusion:**

This study provides normative data on LV and RV parameters in healthy newborns, providing a novel resource for comparison with newborns with structural and functional heart disease.

**Supplementary Information:**

The online version contains supplementary material available at 10.1186/s12968-023-00932-1.

## Introduction

Neonatal cardiovascular magnetic resonance (CMR) is being increasingly utilized for assessment of structural and functional cardiac conditions. CMR has the unique ability to provide a detailed assessment of cardiac anatomy, non-invasive hemodynamics, volumetric data and tissue characterization, without the use of potentially harmful ionizing radiation [1]. In addition, there is a growing body of literature reporting performance of neonatal CMR without anesthesia using the feed-and-wrap technique, suggesting its increasing applicability [2].

In neonates and infants, accurate measurement of biventricular volumes are needed for preoperative assessment and surgical planning for patients with congenital heart disease (CHD) associated with borderline left or right heart structures [3]. While transthoracic echocardiography is often used for initial estimation of ventricular size in neonates, experimental animal studies comparing echocardiography and CMR-derived ventricular volumes have shown CMR to be a more precise method of chamber quantification [4]. Further, measurement of right ventricular (RV) volumes by echocardiography is difficult given the complex tripartite morphology of the chamber with literature now supporting the use of CMR as the preferred method [5, 6]. CMR also has a role in neonates with other heart diseases including tissue characterization of intracardiac masses and pulmonary hypertension [7, 8]. Indeed, CMR is the current gold standard for measurement of biventricular volumes and mass.

The interpretation of CMR in the neonatal population is limited by the inability to compare ventricular volumes and mass to those in normal neonates. There has been a recent momentum in pediatrics to establish CMR datasets for healthy infants and children [9–12], although these studies have minimal inclusion of neonates. Recent work by our group involved performance of CMR in neonates to assess the impact of maternal obesity on cardiac size [13]. However, current literature lacks normative CMR data in a dedicated newborn population. Given this knowledge gap, our study reports normal values of biventricular volumes and mass in a healthy neonatal population using non-sedated free-breathing CMR imaging obtained in the first few days of life.

## Methods

### Study population

Healthy term (37–41 weeks gestation) newborns underwent non-sedated, free-breathing CMR within the first week of life using the ‘feed-and-wrap’ technique. Newborns were a subset of infants born to mothers with a normal body mass index (BMI) in a larger multisite study examining the impact of maternal obesity on newborn outcomes [13, 14]. Neonates born to mothers with a normal BMI [lean cohort, (20–25 kg/m^2^)] in early pregnancy were included to avoid potential effects of maternal obesity. This study was approved by the Regional Ethics Committee and written informed parental consent was obtained. CMR was performed on infants while inpatients on the postnatal ward. No infant had required admission to the intensive care unit.

### Imaging technique

All infants were scanned using acoustic ear protection, pulse oximetry, vector electrocardiogram (ECG) monitoring and without sedation or anesthesia, as described previously [15]. Scans were performed on 3T CMR scanner (Achieva, Philips Healthcare, Best, Netherlands) using an 8-channel pediatric body receive coil. An ECG-gated 2-dimensional balanced steady-state free precession (bSSFP) short axis 10 slice stack optimized for neonatal CMR (acquired in-plane resolution = 1 × 1 mm, slice thickness = 4 mm, TR/TE = 3.8/1.9 ms, flip angle = 35°, signal averages = 4) was placed over the heart, aligned with the mitral valve using previously acquired pilot scans. Four chamber and two chamber cines were additionally included as reference images.

### Post-processing

**Fig. 1:**
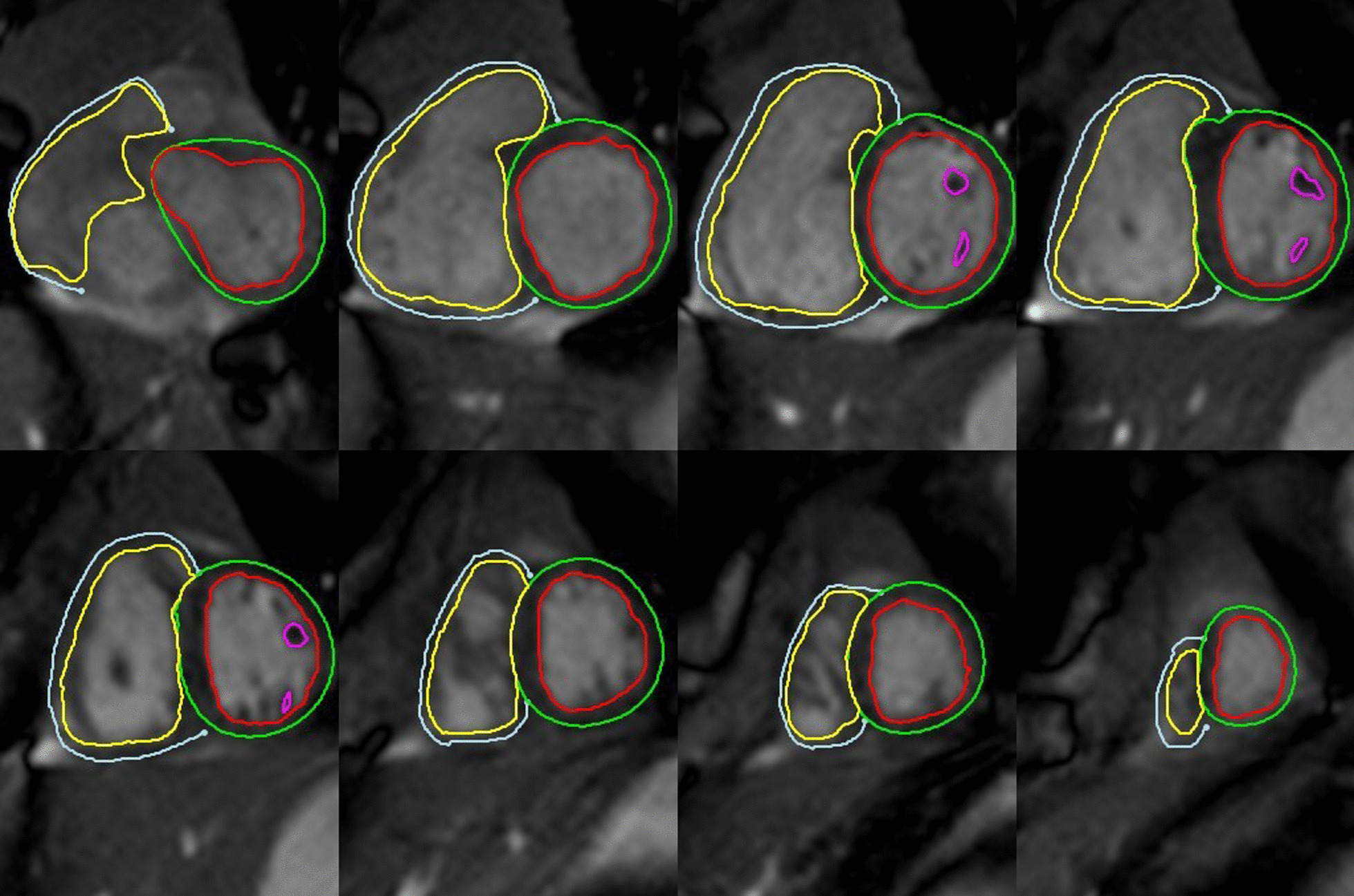
Mulitslice view of short-axis stack in end-diastole with contours. Myocardial borders were defined by performing a detailed manual tracing of the epicardium and endocardium. Left ventricular (LV) papillary muscles were separately contoured and included in the LV myocardial mass (LVM)

Images were transferred to the post-processing Circle Cardiovascular Imaging (cvi42) software (version 5.10.3, Circle Cardiovascular Imaging, Calgary, Canada). End-diastolic and end-systolic phases were determined on the cine bSSFP short-axis stack. Manual contouring of biventricular endocardial borders was performed. Four chamber and two chamber cine images were used for cross-referencing particularly in basal slices for accurate determination of the atrioventricular valvar plane through the cardiac cycle. Left ventricular (LV) papillary muscles were separately contoured and included in the LV mass (LVM) calculation and excluded from the ventricular volume. RV trabeculations and papillary muscles were not contoured separately and were included in RV blood pool. A smooth endocardial border was drawn for the RV volumes. Epicardial borders for both ventricles were traced in diastole to measure the myocardial volume. The interventricular septum, including the septal band was included as part of the LVM. Myocardial volume was multiplied by the factor 1.05 g/ml to obtain the myocardial mass. (Fig. 1) Stroke volume (SV) was calculated as the difference between end-diastolic volume (EDV) and end-systolic volume (ESV). Ejection fraction (EF) was calculated as the SV divided by EDV × 100. LV and RV volumes and mass (RVM) were described as absolute values as well as indexed to weight and body surface area (BSA). RV and LV cardiac outputs were measured by multiplying respective SV by heart rate.

### Statistical analysis

Data are described using mean (standard deviation) for continuous variables and percentages for categorical variables. The volumetric variables were assessed for normal distribution using the Shapiro–Wilk test. The association of gestational age to ventricular volumes was assessed using Pearson correlation coefficient and a p-value of < 0.05 was considered statistically significant. The primary data analysis was performed using Excel 2019 (Microsoft Corporation, Redmond, Washington, USA). The comparative analysis was performed using Stata (version 15.1, Stata Corporation, College Station, Texas, USA).

### Interobserver variability

Ten randomly selected neonates’ CMR data were chosen for interobserver variability assessment. Post-processing was performed on each of these studies by a second independent observer using the same methodology as stated above. Intraclass coefficient (ICC) estimates and their 95% confidence interval (CI) were calculated using Stata (version 15.1; Stata Corporation)***.***

## Results

A total of 20 healthy newborns (65% male) were included in the study. All neonates were born at term with a mean gestational age of 39.9 (1.2) weeks. Average maternal age was 34.2 (5.3) years. Mean age at the time of the scan in hours was 31.8 (14.0) hours (range of 9–56). One newborn did not have height included in the dataset precluding measurement of BSA and indexed values. Demographic details of the cohort are shown in Table 1.

**Table 1:** Demographic variables of the population (n = 20)

Variable	Mean (SD)
Maternal Age (years)	34.2 (5.3)
Maternal BMI (kg/m^2^)	22.0 (1.2)
Gestational age (weeks)	39.9 (1.2)
Age at scan (hours)	31.8 (14.0)
Birth weight (g)	3545 (462)
Length (cm) *	51.9 (3.2)
BSA (m^2^) *	0.23 (0.02)
Heart rate (beats/min)	100.3 (9.8)

*BMI* body mass index, *BSA* body surface area

^*^For these values, n = 19

### Ventricular parameters

The average LV end-diastolic volume (LVEDV) was 9.0 (1.2) ml. The mean LVEDV indexed by BSA (LVEDVI) was 39.0 (4.1) ml/m^2^. The average absolute and BSA indexed LV end-systolic volume (LVESV, LVESVI) was 3.3 (0.5) ml and 14.5 (2.5) ml/m^2^, respectively. LV ejection fraction (LVEF) measured 63.2 (3.4)%. The LVM was 6.0 (0.7) g with an LVM index (LVMI) of 26.4 (2.8) g/m^2^. LVM, LVEDV and LVESV were also indexed to body weight as shown in Table 2.

**Table 2:** Left ventricular (LV) parameters

Variable (unit)	Mean (SD)	Lower/Upper limits **
Absolute values		
LVEDV (ml)	9.0 (1.2)	6.6–11.3
LVESV (ml)	3.3 (0.5)	2.2–4.3
LVEF (%)	63.2 (3.4)	56.4–69.9
LVSV (ml)	5.7 (0.8)	4.0–7.3
LVM (g)	6.0 (0.7)	4.6–7.5
LVCO (ml/min)	624 (110)	404–843
Indexed to body weight		
LVEDV/kg (ml/kg)	2.5 (0.3)	1.9–3.1
LVESV/kg (ml/kg)	0.9 (0.2)	0.5–1.3
LVSV/kg (ml/kg)	1.6 (0.1)	1.4–1.9
LVM/kg (g/kg)	1.7 (0.2)	1.3–2.1
LVCO/kg (ml/min/kg)	177 (25)	126–227
Indexed to BSA		
LVEDV/BSA (ml/m^2^)*	39.0 (4.1)	30.8–47.2
LVESV/BSA (ml/m^2^)*	14.5 (2.5)	9.5–19.4
LVSV/BSA (ml/m^2^)*	24.5 (2.3)	20.0–29.0
LVM/BSA (g/m^2^)*	26.4 (2.8)	20.7–32.0
LVCI (L/min/m2)*	2.7 (0.4)	1.9–3.5

*LVCI* left ventricular cardiac index, *LVCO* left ventricular cardiac output, *LVEDV* left ventricular end-diastolic volume, *LVEF* left ventricular ejection fraction, *LVESV* left ventricular end-systolic volume, *LVM* left ventricular mass, *LVSV* left ventricular stroke volume

*Variables with n = 19, rest of the variables have n = 20, **Calculated as mean ± 2 × SD

Similarly, the mean absolute RV end-diastolic volume (RVEDV) of the cohort was 11.0 (1.7) ml. The RVEDV indexed by BSA (RVEDVI) was 47.4 (4.5) ml/m^2^. The RV ejection fraction (RVEF) was 52.5 (3.3)%. The average RV end-systolic volume (RVESV) was 5.2 (0.9) ml with an RVEDV indexed to BSA (RVEDVI) of 22.6 (2.9) ml/m^2^. Absolute RVM and BSA indexed mass (RVMI) were 2.9 (0.5) g and 12.5 (2.0) g/m^2^, respectively. In addition, the ratio of the RVEDV to the LVEDV was 1.2 (0.2). RV parameters including values indexed to birth weight are shown in Table 3.

**Table 3:** Right ventricular (RV) parameters

Variable (unit)	Mean (SD)	Lower/Upper limits **
Absolute values		
RVEDV (ml)	11.0 (1.7)	7.6–14.4
RVESV (ml)	5.2 (0.9)	3.4–7.0
RVEF (%)	52.5 (3.3)	45.9–59.1
RVSV (ml)	5.8 (0.9)	4.0–7.6
RVM (g)	2.9 (0.5)	1.9–3.9
RVCO (ml/min)	638 (126)	386–890
Indexed to body weight		
RVEDV/kg (ml/kg)	3.1 (0.3)	2.5–3.7
RVESV/kg (ml/kg)	1.5 (0.2)	1.1–1.9
RVSV/kg (ml/kg)	1.6 (0.2)	1.2–2.0
RVM/kg (g/kg)	0.8 (0.1)	0.6–1.0
RVCO/kg (ml/min/kg)	181 (30)	120–241
Indexed to BSA		
RVEDV (ml/ m^2^) *	47.4 (4.5)	38.5–56.4
RVESV (ml/ m^2^) *	22.6 (2.9)	16.9–28.3
RVSV (ml/ m^2^) *	24.9 (2.5)	19.9–29.9
RVM (g/m^2^) *	12.5 (2.0)	8.5–16.5
RVCI (L/min/m2) *	2.7 (0.5)	1.8–3.7

*RVCI* right ventricular cardiac index, *RVCO* right ventricular cardiac output; *RVEDV* right ventricular end-diastolic volume, *RVEF* right ventricular ejection fraction; *RVESV* right ventricular end-systolic volume, *RVM* right ventricular mass, *RVSV* right ventricular stroke volume

*Variables with n = 19, rest of the variables have n = 20

**Calculated as mean ± 2 × standard deviation (SD)

### Biventricular volumes and mass by sex

There were 13 male and 7 female infants. Volumetric data for LV and RV are described for males and females separately in Table 4.

**Table 4:** Ventricular volumes and mass by sex

Variables	Males Mean (SD) N = 13	Females Mean (SD) N = 7
LVEDV (ml)	9.0 (1.0)	8.9 (1.5)
LVESV (ml)	3.4 (0.5)	3.2 (0.6)
LVEF (%)	62.6 (3.5)	64.1 (3.1)
LVSV (ml)	5.6 (0.7)	5.7 (1.0)
LVM (g)	6.0 (0.7)	6.2 (0.8)
RVEDV (ml)	10.9 (1.6)	11.3 (1.9)
RVESV (ml)	5.2 (0.8)	5.2 (1.1)
RVSV (ml)	5.6 (0.9)	6.1 (0.9)
RVEF (%)	51.6 (2.7)	54.1 (3.9)
RVM (g)	2.9 (0.5)	2.8 (0.5)
LVEDV/BSA (ml/m^2^)*	38.9 (3.4)	39.2 (5.7)
LVESV/BSA (ml/m^2^)*	14.6 (2.4)	14.1 (2.8)
LVSV/BSA (ml/m^2^)*	24.3 (1.6)	25.0 (3.4)
LVM/BSA (g/m^2^)*	25.8 (2.3)	27.6 (3.6)
RVEDV/BSA (ml/m^2^)*	46.9 (4.1)	48.7 (5.4)
RVESV/BSA (ml/m^2^)*	22.7 (2.6)	22.4 (3.7)
RVSV/BSA (ml/m^2^)*	24.2 (2.2)	26.3 (2.8)
RVM/BSA (g/m^2^)*	12.7 (2.0)	12.1 (2.1)

*BSA* body surface area, *LVEDV* left ventricular end-diastolic volume, *LVEF* left ventricular ejection fraction, *LVESV* left ventricular end-systolic volume, *LVM* left ventricular mass, *LVSV* left ventricular stroke volume, *RVEDV* right ventricular end-diastolic volume, *RVEF* right ventricular ejection fraction, *RVESV* right ventricular end-systolic volume, *RVM* right ventricular mass, *RVSV* right ventricular stroke volume

*For these values, n = 6 for females

### Relationship to gestational age

There was a weak relationship between LVESVI and gestational age (r = -0.51, p = 0.03). There was no correlation between gestational age and any other variables of biventricular volumes or mass. (Additional file 1: Table S1).

### Interobserver variability

Post-processing was repeated on ten randomly selected newborns by a second independent observer. The intraclass correlation (ICC) estimate between individual measurements and average measurement were calculated. LVEDV, LVESV, RVEDV, RVESV and LVM had excellent inter-observer reproducibility, while that for RVM was slightly lower (Table 5).

**Table 5:** Inter-observer variability testing using intraclass coefficient (ICC) for 10 randomly selected patients with 95% confidence intervals (CI)

Variable	ICC	95% CI
LVEDV	0.98	0.65–1.00
LVESV	0.99	0.98–1.00
LVM	0.97	0.74–0.99
RVEDV	0.98	0.39–1.00
RVESV	0.98	0.24–1.00
RVM	0.94	0.76–0.98

*LVEDV* left ventricular end-diastolic volume, *LVEF* left ventricular ejection fraction, *LVM* left ventricular mass; *RVEDV* right ventricular end-diastolic volume, *RVEF* right ventricular ejection fraction, *RVM* right ventricular mass

## Discussion

Neonatal CMR has historically been challenging primarily due to the need for anesthesia and image optimization. With numerous recent studies reporting refined imaging techniques in neonates without sedation, [15–17] and with the application of CMR data in assessment of patients with CHD, the performance of CMR in this population has increased. There remains, however, a lack of normative data for neonates, although this is available for children and young adults [10]. We believe the present study to be among the first to describe normative data on biventricular chamber volumes, myocardial masses and cardiac output in a cohort of term newborns. This data will enable quantification of z-scores in newborns with structural and functional cardiac lesions.

### Importance of CMR in accuracy of volumetric data

Echocardiography is usually the first line method for assessment of LV volumes and mass. Three dimensional echocardiography is superior to 2-dimensional echocardiography in this regard, although there are wide limits of agreement and it underestimates ventricular volumes when compared to CMR in adult patients [18]. LVM is best estimated by CMR [19]. In an animal model study, bSSFP imaging by CMR correlated well with ventricular mass obtained on autopsy [20]. A correlation between CMR derived LVM and directly measured LVM was also found in studies on human explanted hearts [21]. Furthermore, the RV has a complex geometric tripartite shape that limits its measurement by 2-dimensional echocardiography [5]. CMR is also highly accurate and reproducible in assessment of RV volumes and mass in animal and human studies [22, 23]. Given the superior endocardial definition, lack of radiation and excellent reproducibility associated with CMR, it is currently the gold standard for assessment of biventricular volumes and mass [6, 24].

### Indications for utilization of CMR in neonatal population

Neonatal CMR is being increasingly performed for both CHD and acquired cardiac conditions [8]. Neonates with left sided obstructive lesions such as hypoplastic left heart syndrome, aortic stenosis or Shone’s complex, require accurate determination of LV volumes and mass. Similarly, estimation of RV volumes is important for surgical planning and prognostication in neonates with borderline RV size such as those with unbalanced atrioventricular canal defects and pulmonary atresia. These infants can undergo a univentricular or biventricular surgical procedure. The decision to utilize the hypoplastic ventricle as an independent pumping chamber often involves a complex model that includes measured ventricular volumes. Previously, echocardiographic LV volume was one of the determinants of success of biventricular repair in infants with borderline left-sided structures [25]. However, because these patients often have altered LV geometry and septal configuration 2-dimensional assessment is limited. More recently, CMR-derived ventricular volumes have been included as key determinants of surgical decision making in this population [3]. An indexed LVEDV cutoff of 20 ml/m^2^ or more is used in some centers as favoring biventricular surgery [3, 26]. However, the lack of available CMR normative data as noted by Nathan et al. precludes comparative assessment [26]. Using our dataset, an indexed LVEDV of 20 ml/m^2^ would measure as 4.6 standard deviations below the mean, indicative of moderate LV hypoplasia. In a large multicenter study of patients with pulmonary atresia and intact ventricular septum, a higher baseline RV end-diastolic area measured by 2-dimensional echocardiography was associated with biventricular repair [27]. CMR is likely to play a role in these patients in the future to provide accurate RV measurements. Indications for neonatal CMR, other than CHD include assessment of intracardiac masses, myocardial tissue characterization and pulmonary hypertension [7, 8]. By providing biventricular volumes and mass in healthy newborns, our study provides a reference for neonatal cardiac assessment.

### Image acquisition and analysis

Similar to other centers, neonatal CMR was safely performed without sedation in our study using multi-signal average breathing and a feed-and-wrap technique [2]. Additionally, image-based shimming and frequency stabilization as previously described, were utilized for optimal image quality [15]. Most pediatric studies perform ventriculography using bSSFP short-axis stacks, but others utilize axial stacks [28, 29]. Further, there is heterogeneity in the methodology used for contouring volumes. Some reports identify inclusion of papillary muscles in LV volumes, while others recommend contouring the papillary muscles separately, with inclusion in the LVM calculation [9]. Based on the method used, volumetric data will significantly differ [30]. Our study had excellent interobserver variability for LV parameters but a higher variability for RV parameters. RVM (ICC = 0.94, 95% CI − 0.76–0.98) had the least agreement amongst observers. A lower agreement for RV parameters has similarly been observed by others [9].

### Comparison to literature

Our study showed no association between gestational age and CMR data, although all neonates were born at term within a narrow timeframe (39–41 weeks gestation) which may preclude detection of a significant correlation. LV volumes by CMR in a preterm neonatal population has been evaluated [31]. There have been several efforts in the last decade to assess ventricular volumes and mass in healthy children. A recent study by van der Ven et al. pooled data from healthy children across three European centers to establish normal CMR values in children [12]. The median indexed LVEDV (males, 48 ml/m^2^; females, 51 ml/m^2^) and RVEDV (males, 47 ml/m^2^; females, 54 ml/m^2^) in the youngest cohort reported appear to be higher than the values we observed. However, the cohort grouped children between 0 and 6 years of age (n = 12), likely accounting for the difference. Similarly, a recent large prospective pediatric study was conducted by Olivieri et al. to assess ventricular volumes and mass in healthy children and infants [11]. The study enrolled infants ranging from 21 days to 9 months (n = 23) which reflects a different age group when compared to our study. This further highlights the paucity of normative data in newborns.

## Limitations

Despite being the largest CMR study performed on healthy term newborns, the study has a relatively small sample size (n = 20) that limits detailed analysis. Of note, the study was performed on a 3T scanner. Available adult studies have shown good reproducibility in the volumetric data obtained at 1.5 and 3T, but the lack of supportive pediatric literature potentially limits the generalizability of our data [32]. All neonates included were deemed healthy based on normal newborn clinical examination. However, given that echocardiography was not performed, small shunt lesions (eg. patent ductus arteriosus, patent foramen ovale) or other congenital defects that could affect ventricular volumes and were identified on physical examination, cannot be excluded. Using biventricular SV ratios to calculate Qp:Qs, the mean RV/LV SV of the population is 1.02:1 (range of 0.87: 1.2), supportive of a lack of a significant shunt. It should be noted that all subjects were scanned prior to discharge from hospital and were less than three days of age at the time of the scan. Therefore, these data do not account for normal growth that takes place subsequently in the neonatal period and does not completely represent the neonatal age-group (0–28 days). Further, the use of free-breathing and averaged segmented imaging, particularly in newborns at high heart rates can have an impact on the image quality and measurements. Due to the small sample size and narrow BSA range, allometric normalization, as described in other normative datasets, was not performed [33]. Given that pre-term neonates have distinct clinical and hemodynamic conditions, direct extrapolation of these data to preterm infants is not recommended. Normal values of biventricular volumes and masses for a predominantly pre-term neonatal population have been previously described [17]. Importantly, the cohort described in this study consisted of neonates born to mothers with a normal BMI in early pregnancy and excluded those whose mothers had higher BMI. This could impact on the generalizability of the data given the prevalence of obesity. As our earlier report showed the differences in CMR parameters between infants of lean and obese women, we chose to present data only on the neonates in the “lean group” [13]. Lastly, significant practice variations exist regarding the view (axial versus short-axis) used for ventriculography and the methodology used for post-processing [29]. The methods that we used are in accordance with standards used at our institution. If these data are used as a reference for comparison, we recommend that similar protocols are followed.

## Conclusion

Using non-sedated, free-breathing CMR, we report the largest study of normative data on biventricular volumes and masses in a healthy neonatal population, obtained in the first few days of life. The variables in this study were highly reproducible with good inter-observer agreement. These normative data provide a reference for comparison of ventricular volumes and masses in newborns with structural and functional heart disease.

## Supplementary Information

**Additional file 1: Table S1. **Correlation between gestational age and ventricular variables.

## Data Availability

All data generated or analyzed during this study are included in this published article.

## Abbreviations

BMI: Body mass index
BSA: Body surface area
bSSFP: Balanced steady state free precession
CHD: Congenital heart disease
CMR: Cardiovascular magnetic resonance
ECG: Electrocardiogram
EDV: End-diastolic volume
EF: Ejection fraction
ESV: End-systolic volume
ICC: Intraclass correlation coefficient
IOV: Interobserver variability
LV: Left ventricle/left ventricular
LVCO: Left ventricular cardiac output
LVCI: Left ventricular cardiac index
LVEDV: Left ventricular end-diastolic volume
LVEDVI: Left ventricular end-diastolic volume indexed to BSA
LVEF: Left ventricular ejection fraction
LVESV: Left ventricular end-systolic volume
LVESVI: Left ventricular end-systolic volume indexed to BSA
LVM: Left ventricular mass
LVMI: Left ventricular mass indexed to BSA
LVSV: Left ventricular stroke volume
RV: Right ventricle/right ventricular
RVCO: Right ventricular cardiac output
RVCI: Right ventricular cardiac index
RVEDV: Right ventricular end-diastolic volume
RVEDVI: Right ventricular end-diastolic volume indexed to BSA
RVEF: Right ventricular ejection fraction
RVESV: Right ventricular end-systolic volume
RVESVI: Right ventricular end-systolic volume indexed to BSA
RVM: Right ventricular mass
RVMI: Right ventricular mass indexed to BSA
RVSV: Right ventricular stroke volume
SD: Standard deviation
SV: Stroke volume

## References

[1] Kellenberger CJ, Yoo SJ, Valsangiacomo Büchel ER (2007). Cardiovascular MR imaging in neonates and infants with congenital heart disease. Radiographics.

[2] Windram J, Grosse-Wortmann L, Shariat M, Greer ML, Crawford MW, Yoo SJ (2012). Cardiovascular MRI without sedation or general anesthesia using a feed-and-sleep technique in neonates and infants. Pediatr Radiol.

[3] Grosse-Wortmann L, Yun TJ, Al-Radi O, Kim S, Nii M, Lee KJ (2008). Borderline hypoplasia of the left ventricle in neonates: insights for decision-making from functional assessment with magnetic resonance imaging. J Thorac Cardiovasc Surg.

[4] Heusch A, Koch JA, Krogmann ON, Korbmacher B, Bourgeois M (1999). Volumetric analysis of the right and left ventricle in a porcine heart model: Comparison of three-dimensional echocardiography, magnetic resonance imaging and angiocardiography. Eur J Ultrasound.

[5] Helbing WA, Bosch HG, Maliepaard C, Rebergen SA, van der Geest RJ, Hansen B (1995). Comparison of echocardiographic methods with magnetic resonance imaging for assessment of right ventricular function in children. Am J Cardiol.

[6] Geva T (2014). Is MRI the preferred method for evaluating right ventricular size and function in patients with congenital heart disease?. Circ Cardiovasc Imaging.

[7] Critser PJ, Higano NS, Tkach JA, Olson ES, Spielberg DR, Kingma PS (2020). Cardiac magnetic resonance imaging evaluation of neonatal bronchopulmonary dysplasia–associated pulmonary hypertension. Am J Respir Crit Care Med.

[8] Buechel ERV, Grosse-Wortmann L, Fratz S, Eichhorn J, Sarikouch S, Greil GF (2015). Indications for cardiovascular magnetic resonance in children with congenital and acquired heart disease: An expert consensus paper of the Imaging Working Group of the AEPC and the Cardiovascular Magnetic Resonance Section of the EACVI. Eur Heart J Cardiovasc Imaging.

[9] Buechel EV, Kaiser T, Jackson C, Schmitz A, Kellenberger CJ (2009). Normal right- and left ventricular volumes and myocardial mass in children measured by steady state free precession cardiovascular magnetic resonance. J Cardiovasc Magn Reson.

[10] Kawel-Boehm N, Maceira A, Valsangiacomo-Buechel ER, Vogel-Claussen J, Turkbey EB, Williams R (2015). Normal values for cardiovascular magnetic resonance in adults and children. J Cardiovasc Magn Reson.

[11] Olivieri LJ, Jiang J, Hamann K, Loke YH, Campbell-Washburn A, Xue H (2020). Normal right and left ventricular volumes prospectively obtained from cardiovascular magnetic resonance in awake, healthy, 0–12 year old children. J Cardiovasc Magn Reson.

[12] van der Ven JPG, Sadighy Z, Valsangiacomo Buechel ER, Sarikouch S, Robbers-Visser D, Kellenberger CJ (2020). Multicentre reference values for cardiac magnetic resonance imaging derived ventricular size and function for children aged 0–18 years. Eur Hear J Cardiovasc Imaging..

[13] Groves AM, Price AN, Russell-Webster T, Jhaveri S, Yang Y, Battersby EE (2022). Impact of maternal obesity on neonatal heart rate and cardiac size. Arch Dis Child Fetal Neonatal Ed.

[14] Poston L, Bell R, Croker H, Flynn AC, Godfrey KM, Goff L (2015). Effect of a behavioural intervention in obese pregnant women (the UPBEAT study): a multicentre, randomised controlled trial. Lancet Diabetes Endocrinol.

[15] Price AN, Malik SJ, Broadhouse KM, Finnemore AE, Durighel G, Cox DJ (2013). Neonatal cardiac MRI using prolonged balanced SSFP imaging at 3T with active frequency stabilization. Magn Reson Med.

[16] Tkach JA, Merhar SL, Kline-Fath BM, Pratt RG, Loew WM, Daniels BR (2014). MRI in the neonatal ICU: Initial experience using a small-footprint 1.5-T system. Am J Roentgenol.

[17] Groves AM, Chiesa G, Durighel G, Goldring ST, Fitzpatrick JA, Uribe S (2011). Functional cardiac MRI in preterm and term newborns. Arch Dis Child Fetal Neonatal Ed.

[18] Dorosz JL, Lezotte DC, Weitzenkamp DA, Allen LA, Salcedo EE (2012). Performance of 3-dimensional echocardiography in measuring left ventricular volumes and ejection fraction. J Am Coll Cardiol.

[19] Myerson SG, Bellenger NG, Pennell DJ (2002). Assessment of left ventricular mass by cardiovascular magnetic resonance. Hypertension.

[20] Stephensen SS, Carlsson M, Ugander M, Engblom H, Olivecrona G, Erlinge D (2010). Agreement of left ventricular mass in steady state free precession and delayed enhancement MR images: Implications for quantification of fibrosis in congenital and ischemic heart disease. BMC Med Imaging.

[21] Farber NJ, Reddy ST, Doyle M, Rayarao G, Thompson DV, Olson P (2014). Ex vivo cardiovascular magnetic resonance measurements of right and left ventricular mass compared with direct mass measurement in excised hearts after transplantation: a first human SSFP comparison. J Cardiovasc Magn Reson.

[22] Beygui F, Furber A, Delépine S, Helft G, Metzger JP, Geslin P (2004). Routine breath-hold gradient echo MRI-derived right ventricular mass, volumes and function: accuracy, reproducibility and coherence study. Int J Cardiovasc Imaging.

[23] Koch JA, Poll LW, Godehardt E, Korbmacher B, Mödder U (2000). Right and left ventricular volume measurements in an animal heart model in vitro: first experiences with cardiac MRI at 10 T. Eur Radiol.

[24] Grothues F, Smith GC, Moon JCC, Bellenger NG, Collins P, Klein HU (2002). Comparison of interstudy reproducibility of cardiovascular magnetic resonance with two-dimensional echocardiography in normal subjects and in patients with heart failure or left ventricular hypertrophy. Am J Cardiol.

[25] Schwartz ML, Gauvreau K, Geva T (2001). Predictors of outcome of biventricular repair in infants with multiple left heart obstructive lesions. Circulation.

[26] Nathan M, Emani S, Jsselhof IR, Liu H, Gauvreau K, Del NP (2017). Mid-term outcomes in unbalanced complete atrioventricular septal defect: role of biventricular conversion from single-ventricle palliation. Eur J Cardiothorac Surg.

[27] Maskatia SA, Petit CJ, Travers CD, Goldberg DJ, Rogers LS, Glatz AC (2018). Echocardiographic parameters associated with biventricular circulation and right ventricular growth following right ventricular decompression in patients with pulmonary atresia and intact ventricular septum: results from a multicenter study. Congenit Heart Dis.

[28] Sarikouch S, Schaeffler R, Körperich H, Dongas A, Haas NA, Beerbaum P (2009). Cardiovascular magnetic resonance imaging for intensive care infants: Safe and effective?. Pediatr Cardiol.

[29] Fratz S, Chung T, Greil GF, Samyn MM, Taylor AM, Valsangiacomo Buechel ER (2013). Guidelines and protocols for cardiovascular magnetic resonance in children and adults with congenital heart disease: SCMR expert consensus group on congenital heart disease. J Cardiovasc Magn Reson.

[30] Riffel JH, Schmucker K, Andre F, Ochs M, Hirschberg K, Schaub E (2019). Cardiovascular magnetic resonance of cardiac morphology and function: impact of different strategies of contour drawing and indexing. Clin Res Cardiol.

[31] Broadhouse KM, Finnemore AE, Price AN, Durighel G, Cox DJ, Edwards AD (2014). Cardiovascular magnetic resonance of cardiac function and myocardial mass in preterm infants: a preliminary study of the impact of patent ductus arteriosus. J Cardiovasc Magn Reson.

[32] Maroules CD, McColl R, Khera A, Peshock RM (2008). Interstudy reproducibility of SSFP cine magnetic resonance: impact of magnetic field strength and parallel imaging. J Magn Reson Imaging.

[33] Lopez L, Colan S, Stylianou M, Granger S, Trachtenberg F, Frommelt P (2017). Relationship of echocardiographic z scores adjusted for body surface area to age, sex, race, and ethnicity. Circ Cardiovasc Imaging.

