# Supplementary material for: Normative ranges of biventricular volumes and function in healthy term newborns
Source: J Cardiovasc Magn Reson. 2023 Apr 24;25:26. doi: 10.1186/s12968-023-00932-1 (PMC10127416; doi:10.1186/s12968-023-00932-1)
Supplement: Supplementary file 1 — Additional file 1: Table S1. Correlation between gestational age and ventricular variables. [file 12968_2023_932_MOESM1_ESM.docx]

**Supplemental Table 1: Correlation between gestational age and ventricular variables**

| Variable (unit) | Correlation Coefficient | p-value |
| --- | --- | --- |
| LVEDV (ml) | -0.05 | 0.83 |
| LVESV (ml) | -0.30 | 0.20 |
| LVEF (%) | 0.43 | 0.06 |
| LV mass (g) | -0.05 | 0.83 |
| RVEDV (ml) | 0.05 | 0.85 |
| RVESV (ml) | 0.06 | 0.81 |
| RVEF (%) | -0.02 | 0.95 |
| RV mass (g) | 0.15 | 0.53 |
| LVEDV/BSA* (ml/m2) | -0.39 | 0.09 |
| LVESV/BSA* (ml/m2) | -0.51 | 0.03 |
| RVEDV/BSA*(ml/m2) | -0.18 | 0.45 |
| RVESV/BSA*(ml/m2) | -0.15 | 0.53 |
| LV mass indexed* (g/m2) | -0.37 | 0.12 |
| RV mass indexed*(g/m2) | -0.05 | 0.84 |
| **For these values, n=19* | | |

LVEDV = left ventricular end-diastolic volume; LVEF = left ventricular ejection fraction; LVESV = left

ventricular end-systolic volume; LVM = left ventricular mass; RVEDV = right ventricular end-diastolic

volume; RVEF = right ventricular ejection fraction; RVESV = right ventricular end-systolic volume; RVM = right ventricular mass.
